# Mitochondrial ROS accumulation inhibiting JAK2/STAT3 pathway is a critical modulator of CYT997-induced autophagy and apoptosis in gastric cancer

**DOI:** 10.1186/s13046-020-01621-y

**Published:** 2020-06-23

**Authors:** Ya Cao, Jinglong Wang, Hua Tian, Guo-Hui Fu

**Affiliations:** 1 Pathology Center, Shanghai General Hospital/Faculty of Basic Medicine, Key Laboratory of Cell Differentiation and Apoptosis of Chinese Ministry of Education, Institutes of Medical Sciences, Shanghai Key Laboratory of Gastric Neoplasms, Shanghai Institute of Digestive Surgery, Ruijin Hospital, Shanghai Jiao Tong University School of Medicine, No. 280, South Chong-Qing Road, Shanghai, 200025 China; 2 State Key Laboratory of Oncogenes and Related Genes, Shanghai Cancer Institute, Renji Hospital, Shanghai Jiaotong University School of Medicine, 25/Ln 2200, Xietu Road, Shanghai, 200032 China

**Keywords:** CYT997, ROS, JAK2/STAT3, Apoptosis, Gastric cancer

## Abstract

**Background:**

Gastric cancer (GC) is a common form of malignant cancer in worldwide which has a poor prognosis. Despite recent improvements in the treatment of GC, the prognosis is not yet satisfactory for GC patients*.* CYT997, a novel microtubule-targeting agent, recently has been identified to be a promising anticancer candidate for the treatment of cancers; however, the effects of CYT997 in GC remain largely unknown.

**Methods:**

Cell proliferation and apoptosis were detected by CCK8 assay and flow cytometry. The mitochondrial ROS were detected by confocal microscope and flow cytometry. Gastric cancer patient-derived xenograft (PDX) model was used to evaluate its antitumor activity of CYT997 in vivo.

**Results:**

CYT997 inhibited gastric cancer cell proliferation and induced cell apoptosis and triggered autophagy. CYT997 induced apoptosis through triggering intracellular mitochondrial ROS generation in GC cells. ROS scavengers N-acetylcysteine (NAC) and Mitoquinone (MitoQ) distinctly weakened CYT997-induced cell cycle G2/M arrest and apoptosis in GC cells. Pretreatment with autophagy inhibitor 3-MA promoted the effect of CYT997 on cells apoptosis. Mechanistically, CYT997 performed its function through regulation of Janus kinase 2 (JAK2)/signal transducer and activator of transcription 3 (STAT3) signaling pathway in GC cells. In addition, CYT997 inhibited growth of gastric cancer patient-derived xenograft **(**PDX) tumors.

**Conclusions:**

CYT997 induces autophagy and apoptosis in gastric cancer by triggering mitochondrial ROS accumulation to silence JAK2/STAT3 pathway. CYT997 might be a potential antitumor drug candidate to treat GC.

## Introduction

Gastric cancer (GC) is the third leading cause of cancer-related deaths and the fifth most common malignancy in worldwide [1, 2]. The 5-year survival rate of GC largely depends on clinical stage, ranging between 10 and 93% [2, 3]. Patients with GC are often treated with surgery and/or chemotherapy according to the patients’ specific condition, but recurrence and metastasis are usually common and prognosis is often poor [4, 5]. Chemotherapy is still the main treatment for advanced GC. Therefore, finding new drugs is urgent for the treatment of patients with GC.

Microtubules participate in many biological processes in cells, such as maintenance of cell shape, cell motility and mitosis. Disrupting microtubules’ function can affect the spindle checkpoint and cell cycle progression, resulting in cell death [6, 7]. So, targeting microtubules, such as paclitaxel, vinblastine and docetaxel, are efficient strategies for cancer treatment and have been used to treat different types of human cancers [8]. However, they still have substantial defects such as lack of oral bioavailability, narrow therapeutic windows, potential side effects and cardiovascular events in clinical chemotherapy [9]. To overcome these problems, it’s urgent to explore novel microtubule-targeting agents. CYT997 is a new microtubule-targeting agent selected by Cytopia’s small molecule library and has been proved to have anti-tumor functions by damaging cellular microtubules and preventing tubulin polymerization [10, 11]. It also has been studied in phase I clinical trials that CYT997 had vascular disrupting activity and potent cytotoxicity in several cancers, including pancreatic adenocarcinoma, non-small cell lung cancer, breast cancer and colorectal cancer. Therefore, it might optimally be performed in anti-cancer therapeutics [12, 13].

Reactive oxygen species (ROS), active forms of oxygen, have toxic effects on various cells. ROS play an important role in tumorigenesis and progression [14]. ROS have been targeted by a number of anticancer drugs. Antitumor drugs anthracyclines and topoisomerase inhibitors such as doxorubicin, adriamycin, daunorubicin, and epirubicin can block DNA synthesis, topoisomerase II activity and complex I/II and increase mitochondrial ROS production to kill tumor cells [14, 15]. Platinum-based drugs including cisplatin, carboplatin and oxaliplatin also can induce tumor cell death by maintaining very high levels of ROS [16, 17]. Therefore, ROS should be exploited as a therapeutic target to inhibit tumor growth.

Previous studies have shown that CYT997 inhibited the proliferation of many types of tumors. For example, in acute myeloid leukemia, CYT997 killed acute myeloid leukemia cells via activation of caspases and inhibition of PI3K/Akt/mTOR pathway [18]. Teng et al. also reported that CYT997 inhibited proliferation and invasion of prostate cancer cells by inhibiting Src activity [19]. In addition, CYT997 induced cells death by enhancing ER stress in osteosarcoma [20]. Although these researches provided the mechanisms of the anticancer activity of CYT997, the effects and molecular mechanism of CYT997 in GC remain unclear. In this study, we explored the effects of CYT997 on the proliferation of GC cells as well as the underlying molecular mechanisms of these processes.

## Materials and methods

### Cell lines, primary gastric cancer cells and cell culture

Human GC cell lines SGC-7901, MKN45, AGS, and BGC-823 were purchased from the Cell Bank of the Shanghai Institute for Biological Science (Shanghai, China). All cells were cultured in RPMI-1640 (Hyclone, Thermo Fisher, USA) medium with 10% fetal bovine serum (FBS) (Hyclone). The cells were maintained at 37 °C in a humidified incubator with 5% CO_2_.

The fresh GC tumor tissue from GC patient was acquired and washed three times with PBS containing 1% penicillin/streptomycin (Invitrogen, Carlsbad, CA, USA), then, dissociated as small as possible with scissors, digested with collagenase IV (Sigma), 90 min at 37 °C, stopped digestion and centrifugated with 1000 rpm, 3 min, finally, resuspended and cultured with DMEM/F12 (Hyclone) medium containing 10% FBS and 1% penicillin/streptomycin.

### Reagents and antibodies

CYT997 (MF: C24H30N6O2, MW: 434.53, purity: 99.46%), IL-6 and Mitoquinone (MitoQ) were bought from MCE (Shanghai, China). 3-methyladenine (3-MA) and N-acetylcysteine (NAC) were obtained from Sigma-Aldrich. GAPDH, Cyclin B1, p21, PARP, cleaved PARP, caspase 3, cleaved caspase 3, LC3B, Beclin-1, phosphorylated JAK2 (p-JAK2), JAK2, phosphorylated STAT3^(Tyr705)^(p-STAT3), STAT3, Bcl-2, Survivin, Cyclin D1 and PCNA antibodies were purchased from Cell Signaling Technology (Beverly, MA, USA).

### Cell viability and colony formation assay

Cell proliferation was performed with a CCK-8 kit (Dojindo, Tokyo, Japan). Cells (1× 10^4^) were seeded in 96-well plates. After 6 h, they were treated with different concentrations of CYT997. The OD value was measured at a wavelength of 450 nm. For colony formation assays, 500 cells were plated in each well of a 6-well plate, cultured in RPMI-1640 medium with 10% FBS. CYT997 was added in each well for about 14 days until the cells become visible colonies. Colonies were fixed with 4% paraformaldehyde and stained with crystal violet for 15 min at room temperature. Each experiment was performed in triplicate.

### Cell cycle and apoptosis analysis

Cells were seeded in six-well plates, pretreated with serum starvation for 12 h, then treated with CYT997 for 12 h. For cell cycle analysis, the cells were collected, fixed in 75% ethanol at 4 °C overnight and stained with PI/RNase staining buffer for 15 min. For apoptosis analysis, cells were collected, washed twice with cold PBS and resuspended in binding buffer containing Annexin V-FITC and PI (Invitrogen Life Technologies, Carlsbad, CA, USA). The cell cycle and apoptosis analyses were performed on the Accuri C6 (BD Biosciences, Mountain View, CA, USA) and the data were analyzed by ModFit LT software (FACSCalibur).

### Western blotting analysis

Samples including cells and tissues were lysed in ice-cold RIPA with phenylmethylsulfonyl fluoride and protease inhibitor (Thermo Scientific, Fremont, CA, USA) for 30 min. Lysates were collected, centrifuged and the supernatant was collected. The concentration of protein was measured by a Pierce BCA protein assay kit (Thermo Scientific, Fremont, CA, USA). Equal amounts of protein were separated by SDS-PAGE and transferred to polyvinylidene difluoride (PVDF) membrane (Millipore, Billerica, MA, USA). The membranes were blocked in 5% non-fat milk at room temperature for 1 h and then incubated with specific primary antibodies at 4 °C overnight, washed 3 times by TBST, and then incubated with secondary antibodies for 1 h. Signals were detected by enhanced chemiluminescence kit (Millipore, Billerica, MA, USA).

### Measurement of intracellular ROS and mitochondrial superoxide

Intracellular ROS production was measured by using the Reactive Oxygen Species Assay Kit (Beyotime, Shanghai, China). Cells were seeded in six-well plates overnight and exposed to CYT997 for 12 h.Then, cells were collected, incubated with 10 μM DCFH-DA for 30 min in the dark and washed 3 times. The level of ROS was determined by fluorescence microscopy (Leica, Wetzlar, Germany) and flow cytometry (BD Biosciences; San Jose, CA, USA). Through using MitoSOX Red dye (Invitrogen), mitochondrial superoxide level was detected.

### Mitochondrial membrane potential detection

JC-1 Assay Kit (Beyotime, Jiangsu, China) was used to measure the mitochondrial membrane potential according to the manufacturer’s instructions. Briefly, cells after treatment with CYT997 (50 nM) for 12 h, stained with JC-1 for 20 min at 37 °C and then analyzed by a confocal laser scanning microscope and flow cytometry.

### GFP-LC3 puncta assay and LysoTracker red staining

Cells were transiently transfected with GFP-LC3 plasmid using Lipofectamine 3000 (Invitrogen, Carlsbad, CA, USA) according to the manufacturer’s protocol. After 24 h, cells were treated with 50 nM CYT997 for 24 h.Then cells were washed with PBS, incubated with 50 nM of LysoTracker Red DND-99 (Invitrogen) in the dark for 30 min, washed with PBS again, fixed with 4% paraformaldehyde for 15 min and incubated with 4′,6-diamidino-2-phenylindole (DAPI) for 5 min. Images were obtained by using a confocal laser scanning microscope (Leica, Germany).

### Plasmid construction and transfection

The coding genes of human STAT3 and JAK2 were cloned into the mammalian expression vector pcDNA3.1. SGC-7901 cells were transiently transfected with the pcDNA3.1-STAT3 plasmid and pcDNA3.1-JAK2 plasmid through using Lipofectamine 3000 according to the manufacturer’s instruction (Invitrogen, Gaithersburg, MD, USA).

### GC patient-derived xenograft (PDX) mice experiment

The GC tumor tissue from a GC patient was acquired and kept in culture medium on ice for engraftment within 2 h of resection. Then, the tissues were washed with PBS three times. A piece of about 5mm^3^ tissue was cut and implanted subcutaneously into the flank region of six-week-old BALB/c nude mice by using a trocar. Female athymic BALB/c nude mice (6–8 weeks old) were purchased from Shanghai Experimental Animal Center, Chinese Academy of Science. Until the tumors reached ~ 50 mm^3^, the mice were randomly assigned to two groups: (1) control group (*n* = 8), injected intraperitoneally with normal saline (NS). (2) treated group (n = 8): injected intraperitoneally with CYT997 (15 mg/kg) every day. The experiments were approved by the animal research committee in Shanghai Jiao Tong University.

### Immunohistochemistry (IHC)

For immunohistochemical staining, slides were deparaffinized, rehydrated, incubated in 3% H_2_O_2_ to block endogenous peroxidase activity. After antigen retrieval processed by boiling in sodium citrate for 30 min, slides were blocked by using 10% goat serum for 15 min, followed by incubation with specific primary antibodies at 4 °C overnight. Primary tumor samples were immunostained with p-STAT3 (1:50), PCNA (1:3000), cleaved caspase 3 (1:50) and LC3B (1:500). Then slides were washed 3 times, incubated with the second antibody at room temperature for 30 min, washed 3 times and incubated with diaminobenizidine (DAB) for 3 min. Finally, the nuclei were counterstained with Mayer’s hematoxylin.

### Statistical analysis

Statistical analyses were performed using SPSS 19.0 software (IBM Corporation, Chicago, USA). Student’s t-test was used and all data were presented as mean ± SD. *P*-values< 0.05 were considered as statistically significant.

## Results

### CYT997 inhibits GC cells proliferation and induces apoptosis

**Fig. 1:**
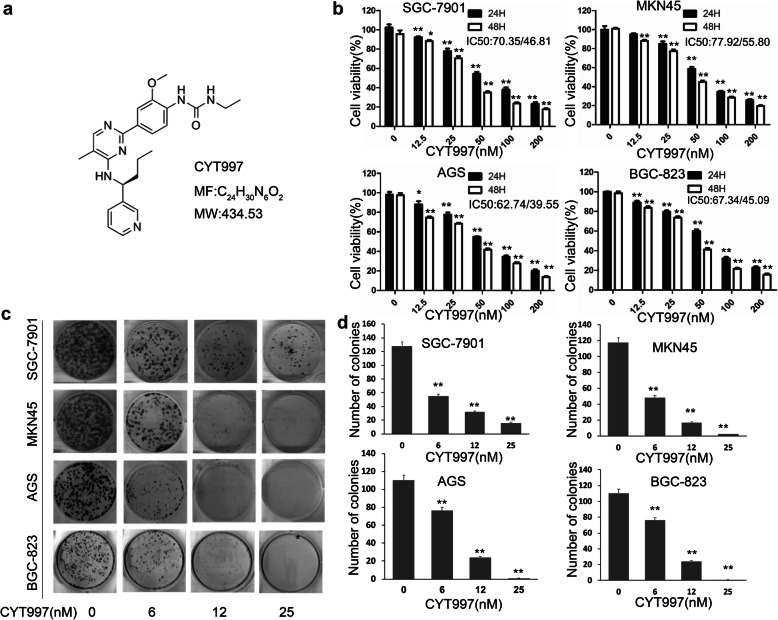
CYT997 inhibited proliferation and colony formation of GC cells. **a** Chemical structural of CYT997. **b**-**d** SGC-7901, MKN45, AGS and BGC-823 cells were treated with CYT997 for 24 and 48 h. Cell proliferation was detected by a CCK8 assay **b** and a colony formation assay **c** and **d**. *p < 0.05; **p < 0.01

**Fig. 2:**
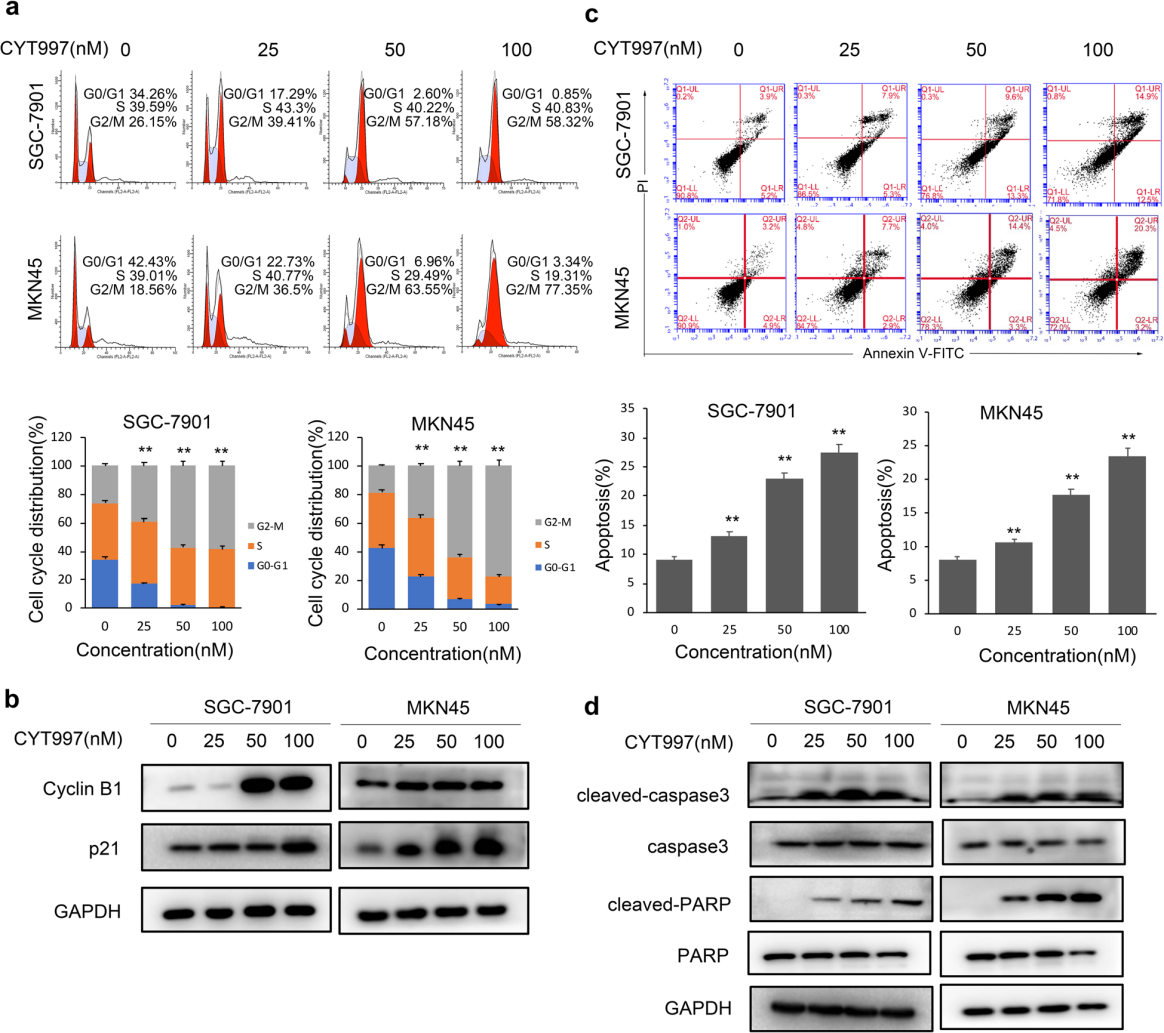
CYT997 induced cell cycle G2/M arrest and apoptosis in GC cells. **a** The cell cycle distribution of SGC-7910 and MKN45 cells treated with CYT997 was analyzed by flow cytometry.**p < 0.01. **b** Expression of Cyclin B1 and p21 was detected by western blotting in SGC-7901 and MKN45 cells treated with CYT997. **c** Following treatment of SGC-7901 and MKN45 cells in different dose of CYT997 for 24 h, apoptotic cells were detected by Annexin V-FITC and PI double staining. **p < 0.01.**d** Expression of cleaved caspase 3, caspase 3, cleaved PARP and PARP was detected by western blotting in SGC-7901 and MKN45 cells treated with CYT997

To explore the effects of CYT997 (Fig.1a) on the proliferation of GC cells, human GC cells SGC-7901, MKN45, AGS, and BGC-823 were treated with CYT997 for 24 h and 48 h. Cell viability was detected by CCK8 assay. CYT997 inhibited proliferation of GC cells in a dose- and time-dependent manner (Fig. 1b). The IC50 values of GC cells SGC-7901, MKN45, AGS, and BGC-823 were 70.35, 77.92, 62.74 and 67.34 nM, respectively, at 24 h and 46.81, 55.80, 39.55 and 45.09 nM, respectively, at 48 h. We also chose osteosarcoma cell 143B as a control reference cell line.143B cells were treated with CYT997 for 24 h and 48 h. The IC50 values were 88.7 nM and 64.8 nM (Additional file 1: Fig. S1). Colony formation assay also showed that the colony formation ability of GC cells was inhibited in the CYT997-treated cells (Fig. 1c and d). To further investigate the mechanism of CYT997-induced suppression of GC cell proliferation, the cell cycle distributions of SGC-7901 and MKN45 cells were determined by flow cytometry. Our results showed that CYT997 induced G2/M arrest in SGC-7901 and MKN45 cells (Fig. 2a). Furthermore, cell cycle-related proteins Cyclin B1 and p21 were upregulated in CYT997 treated GC cells (Fig. 2b; Additional file 1: Fig. S2a).

Apoptosis is also widely believed to be the major antiproliferative mechanism of anticancer drugs in many tumor cell types. Therefore, we also investigated the effect of CYT997 on cell apoptosis in GC cells. Our results showed that CYT997 induced apoptosis in SGC-7901 and MKN45 cells in a dose-dependent manner (Fig. 2c). Furthermore, apoptosis-related protein cleaved caspase 3 and cleaved PARP were significantly increased in GC cells treated with CYT997 (Fig. 2d; Additional file 1: Fig. S2b). Therefore, these results suggested that CYT997 inhibited cell proliferation and induced apoptosis in the GC cells.

### CYT997 triggers protective autophagy in GC cells

**Fig. 3:**
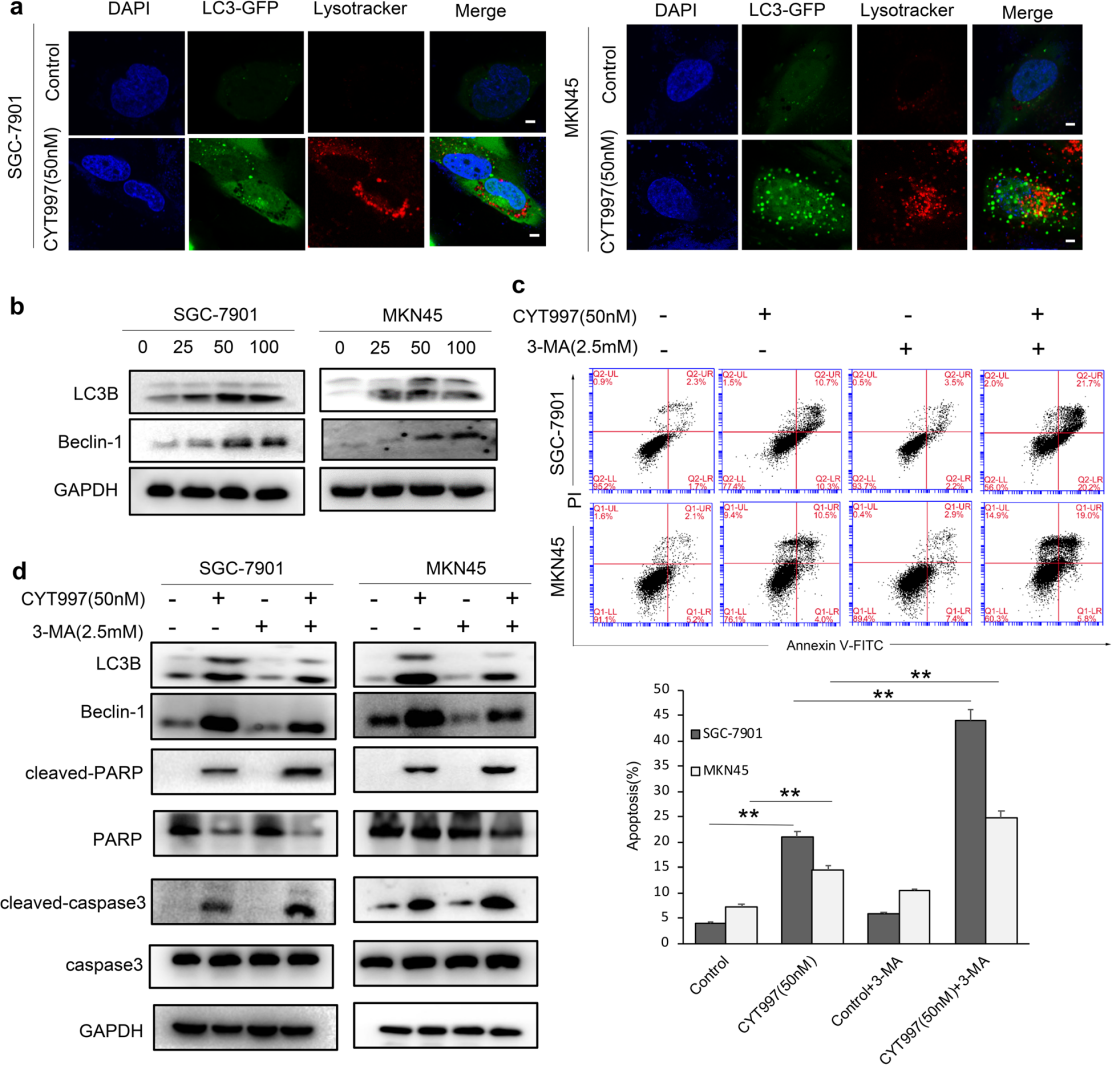
CYT997 triggered autophagy in SGC-7901 and MKN45 cells. **a** After transient transfection with the GFP-fused LC3 plasmid, SGC-7901 and MKN45 cells were treated with CYT997 for 24 h. Cells were incubated with LysoTracker Red DND-99. The distribution of GFP-LC3B expression and LysoTracker Red was visualized by confocal microscopy. Scale bars = 7.5 μm. Results were validated by three separate experiments. **b** Expression of LC3B and Beclin-1 was detected by western blotting in SGC-7901 and MKN45 cells treated with CYT997. **c** SGC-7901 and MKN45 cells were treated with CYT997 alone or in combination with 3-MA. Cell apoptosis was detected by flow cytometry. **p < 0.01.**d** Expression of cleaved PARP, PARP, cleaved caspase 3 and caspase 3 was detected by western blotting in SGC-7901 and MKN45 cells treated with CYT997 alone or in combination with 3-MA

Autophagy is a catabolic process in which autophagosomes is formed, and it has double-sided effect in cancer proliferation and death [21, 22]. LC3 and Beclin-1 are key molecular regulator of autophagy and can be used as autophagy marker [23, 24]. To investigate whether CYT997 could trigger autophagy in GC cells, Lyso-Tracker Red, a membrane acidotropic dye probe was used to mark cellular acidic organelles (AVOs), such as lysosomes and autolysosomes. We found that SGC-7901 and MKN45 cells treated with CYT997 showed more AVOs in cytoplasm than control cells (Fig. 3a). Then, GFP-LC3 puncta transfection way was applied to analyze the formation of autophagosomes. The results showed that SGC-7901 and MKN45 cells treated with CYT997 exhibited significantly higher numbers of GFP-LC3 puncta than control cells (Fig. 3a; Additional file 1: Fig. S3a-3b). Additionally, we found that expression of Beclin-1 were upregulated in GC cells treated with CYT997 in a dose-dependent manner (Fig. 3b; Additional file 1: Fig. S3c). To further explore the role of autophagy in CYT997-treated GC cells, SGC-7901 and MKN45 cells were pretreated with autophagy inhibitor 3-MA prior to CYT997 treatment. The outcomes displayed that pretreatment with 3-MA promoted the effect of CYT997 on cell apoptosis (Fig. 3c). Furthermore, the expression of LC3B and Beclin-1 were decreased, while cleaved caspase 3 and cleaved PARP were increased in GC cells (Fig. 3d; Additional file 1: Fig. S3d). Collectively, autophagy may have additional cytoprotective effects by protecting cells against effects of CYT997.

### CYT997 induces mitochondrial ROS generation in GC cells

**Fig. 4:**
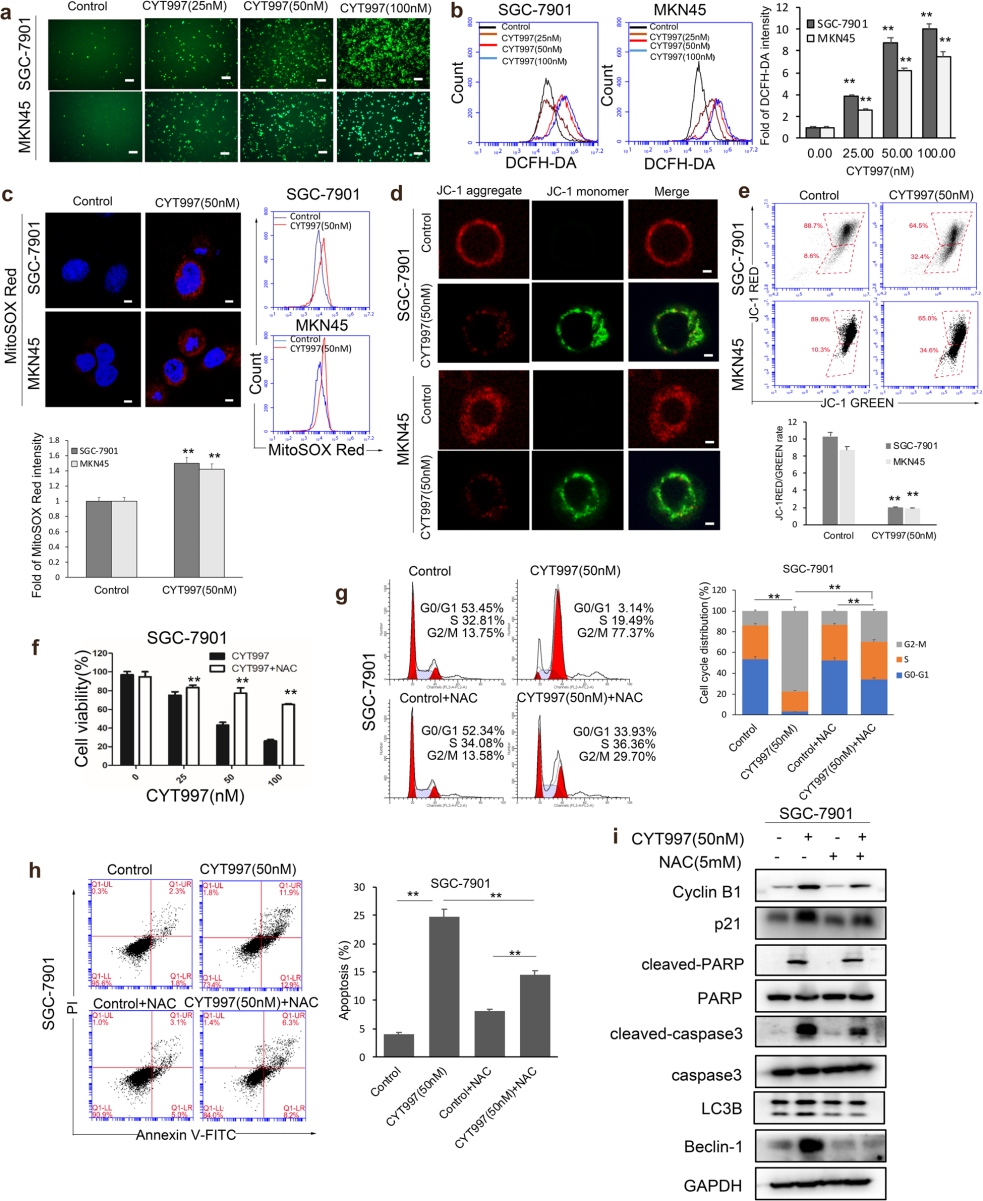
CYT997 induced apoptosis through triggering intracellular mitochondrial ROS generation in SGC-7901 and MKN45 cells. **a** The level of ROS was detected in SGC-7901 and MKN45 cells treated with CYT997 by fluorescence microscopy. Scale bars = 50 μm. **b** ROS levels of SGC-7901 and MKN45 cells treated with CYT997 were determined by flow cytometry. The fold change in DCFH-DA intensity was shown in the right of bar. **p < 0.01. **c** Mitochondrial ROS stained with MitoSOX Red dye were detected by confocal microscope and flow cytometry. The fold change in MitoSOX Red intensity was shown in the right bar. Scale bars = 7.5 μm. **d**-**e** The mitochondrial membrane potential stained with the fluorescent mitochondrial probe JC-1 was measured and assessed by confocal microscope **d** and flow cytometry **e**. Scale bars = 5 μm.**f** SGC-7901 cells were treated with CYT997 alone or in combination with NAC (5 mM). Cell viability was detected by a CCK8 assay. **p < 0.01.**g** The cell cycle distribution of SGC-7910 cells treated with CYT997 alone or in combination with NAC (5 mM) was analyzed by flow cytometry. **p < 0.01. **h** SGC-7901 cells were treated with CYT997 alone or in combination with NAC (5 mM). Apoptosis was detected by flow cytometry. ** *p* < 0.01. **i** Expression of cell cycle, autophagy and apoptosis related proteins was detected by western blotting in SGC-7901 cells

**Fig. 5:**
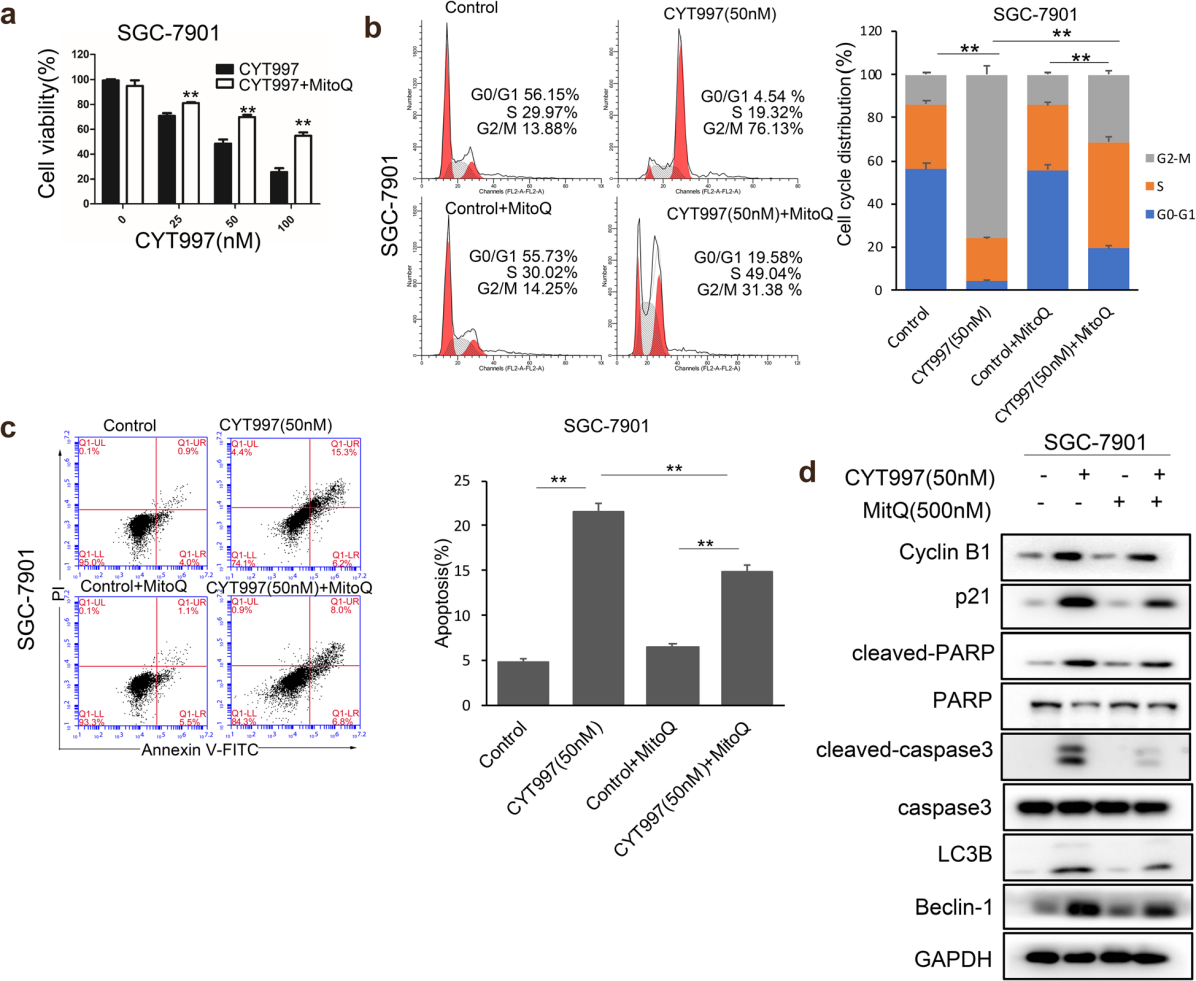
Mitochondrial ROS inhibitor MitoQ reversed the inhibition of CYT997 in GC cells. **a** SGC-7901 cells were treated with CYT997 alone or in combination with MitoQ (500 nM). Cell viability was detected by a CCK8 assay. **b** The cell cycle distribution of SGC-7901 cells treated with CYT997 alone or in combination with MitoQ (500 nM) was analyzed by flow cytometry. **c** SGC-7901 cells were treated with CYT997 alone or in combination with MitoQ (500 nM). Apoptosis was detected by flow cytometry. **d** Expression of cell cycle, autophagy and apoptosis related proteins was detected by western blotting in SGC-7901 cells

Reactive oxygen species (ROS) are critical regulators of apoptosis [25]. Many chemotherapeutic agents induce tumor cells apoptosis by initially inducing cells to accumulate ROS [26, 27]. To clarify whether CYT997-induced apoptosis was due to ROS production, ROS assay was applied to detect ROS level in GC cells. Our results showed that SGC-7901 and MKN45 cells treated with CYT997 exhibited a distinct enhancement in the DCFH-DA fluorescent signal than control (Fig. 4a and b). These results indicated that CYT997 could increase ROS production in GC cell. To explore the source of ROS, MitoSOX Red dye was used to detect mitochondrial ROS by confocal microscope and flow cytometry. We found that SGC-7901 and MKN45 cells treated with CYT997 showed an obvious improvement in the MitoSOX Red fluorescent signal than control (Fig. 4c). These results indicated that CYT997 increased the production of mitochondrial ROS in GC cells. To further prove mitochondrial ROS, JC-1 assay was used to detect the mitochondrial membrane potential by confocal microscope and flow cytometry. Our results showed that red fluorescent signal was significantly attenuated, while green fluorescent signal was notably enhanced in cells treated with CYT997. The JC-1 red/green fluorescence intensity ratio was distinctly reduced in GC cells treated with CYT997 (Fig. 4d and e). Mitochondrial dysfunction and imbalance often lead to mitochondrial membrane potential change and trigger mitochondrial ROS release [28, 29]. The results suggested that GC cells mitochondrial membrane potential was decreased after CYT997 treatment and mitochondrial function was damage, which increased mitochondrial ROS production. Next, ROS scavengers NAC and MitoQ were used to investigate the effects of antioxidants on CYT997-induced cell apoptosis. The results showed that NAC and MitoQ obviously attenuated the CYT997-induced proliferation inhibition (Fig. 4f and 5a). NAC and MitoQ also distinctly inhibited CYT997-induced cell cycle G2/M arrest and apoptosis in GC cells (Fig. 4g, h, 5b, c). Furthermore, the expression of Cyclin B1, p21, cleaved caspase 3, cleaved PARP, LC3B, Beclin-1 was downregulated in cells pretreated with NAC or MitoQ than only treated with CYT997 (Fig. 4i, 5d; Additional file 1: Fig. S4 and S5a). Therefore, these data indicated that CYT997 could induce apoptosis in GC cells through enhancing mitochondrial ROS.

### CYT997 inhibits cell proliferation and induces apoptosis by regulating of JAk2/STAT3 pathway in GC cells

**Fig. 6:**
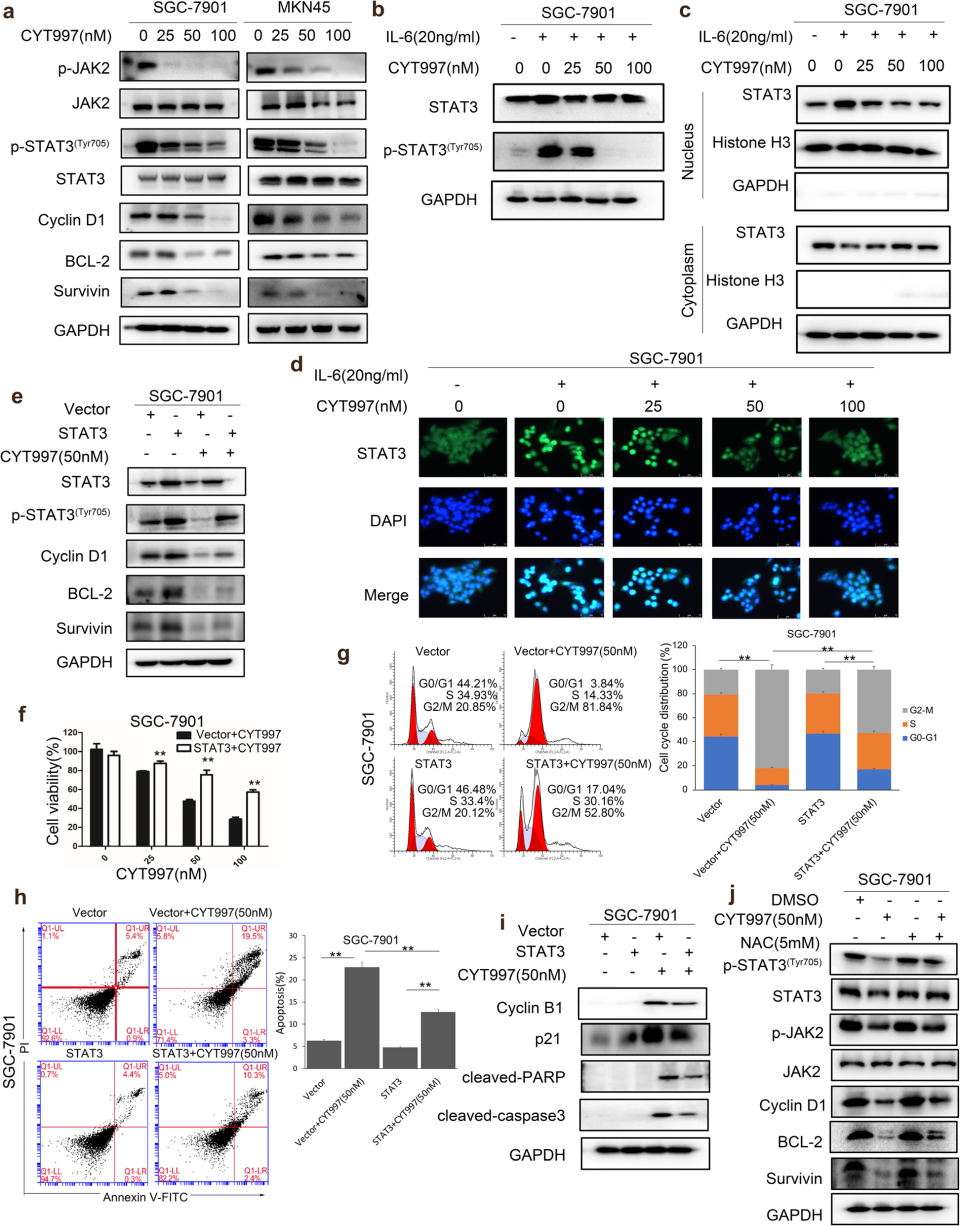
CYT997 performs its function through regulation of STAT3 signaling pathway in SGC-7901 and MKN45 cells. **a** Expression of phosphorylated STAT3^(Tyr705)^ (p-STAT3), phosphorylated JAK2 (p-JAK2) and the major downstream protein Bcl-2, Survivin and Cyclin D1 was detected by western blotting in SGC-7901 and MKN45 cells treated with CYT997. **b** SGC-7901cells were treated with CYT997 or in combination with IL-6. The expression of p-STAT3 and STAT3 was detected by western blotting in SGC-7901 cells. **c**-**d** SGC-7901 cells were treated with CYT997 or in combination with IL-6. The cytoplasmic and nuclear distribution of STAT3 was detected by western blotting **c** and fluorescent microscope **d** in SGC-7901 cells. **e**-**h** SGC-7901 cells were transfected with STAT3 vector, and then treated with CYT997. The expression of p-STAT3, STAT3, Cyclin D1, Bcl-2 and Survivin was detected by western blotting **e**. Cell viability was detected by a CCK8 assay **f**. The cell cycle distribution was analyzed by flow cytometry **g**. Apoptosis was detected by flow cytometry **h**. **i** SGC-7901 cells transfected with STAT3 vector were treated with CYT997. The expression of Cyclin B1, p21, cleaved PARP and cleaved caspase 3 was detected by western blotting. **j** SGC-7901 cells were treated with CYT997 or in combination with NAC. The expression of p-STAT3, STAT3, p-JAK2, JAK2, Cyclin D1, Bcl-2, and Survivin was detected by western blotting in SGC-7901 cells

ROS modulate various cell signaling pathways including STAT3. STAT3, as a transcription factor, plays an important role in tumor cell proliferation and progression [30]. Therefore, we wondered if CYT997 could affect the JAK/STAT3 pathway in GC cells. Our results showed that p-STAT3 was inhibited in CYT997 treated cells. Moreover, p-JAK2 was also inhibited by CYT997, while the JAK2 did not change. Additionally, the major downstream targets of STAT3 were inhibited by CYT997, including Bcl-2, Survivin and Cyclin D1 (Fig. 6a; Additional file 1: Fig. S6a). Cytokine IL-6, a major mediator of inflammation and activator of STAT3, serves to block apoptosis and promote tumor survival. We found that activation of STAT3 induced by IL-6 was reversed by CYT997 (Fig. 6b; Additional file 1: Fig. S6b). Furthermore, IL-6-induced nuclear accumulation of STAT3 was reversed by CYT997 using western blotting and immunofluorescence assay (Fig. 6c and d; Additional file 1: Fig. S6c).

To further certify the molecular mechanism of CYT997, STAT3 and JAK2 plasmid were transfected into SGC-7901 cells respectively. We found that p-STAT3 and its downstream protein were activated by STAT3 overexpression. Overexpression of STAT3 weakened the effects of CYT997 on the expression of p-STAT3, Cyclin D1, Bcl-2 and Survivin in SGC-7901 cells (Fig. 6e; Additional file 1: Fig. S7a). CYT997-induced cell proliferation inhibition, cell cycle arrest and apoptosis were attenuated by STAT3 overexpression in SGC-7901 cells (Fig.6f-h). The expression of Cyclin B1, p21, cleaved caspase 3 and cleaved PARP was partly reversed by STAT3 overexpression (Fig. 6i; Additional file 1: Fig. S7b). The similar results were further proved by JAK2 overexpression (Additional file 1: Fig. S8). Therefore, all results indicated that CYT997 inhibited GC cell proliferation by regulating of JAK2/STAT3 pathway.

Next, we explored whether CYT997 affected the JAK2/STAT3 pathway through ROS generation in GC cells. CYT997-induced inhibition of p-JAK2, p-STAT3, Bcl-2, Survivin and Cyclin D1 was reversed by NAC in SCG-7901 cells (Fig. 6j; Additional file 1: Fig. S7c). In addition, the similar results were found using MitoQ (Additional file 1: Fig. S5b). Collectively, these results suggested that CYT997 inhibited JAK2/STAT3 pathway through ROS generation in GC cells.

### CYT997 suppresses in vivo growth of PDX tumors

**Fig. 7:**
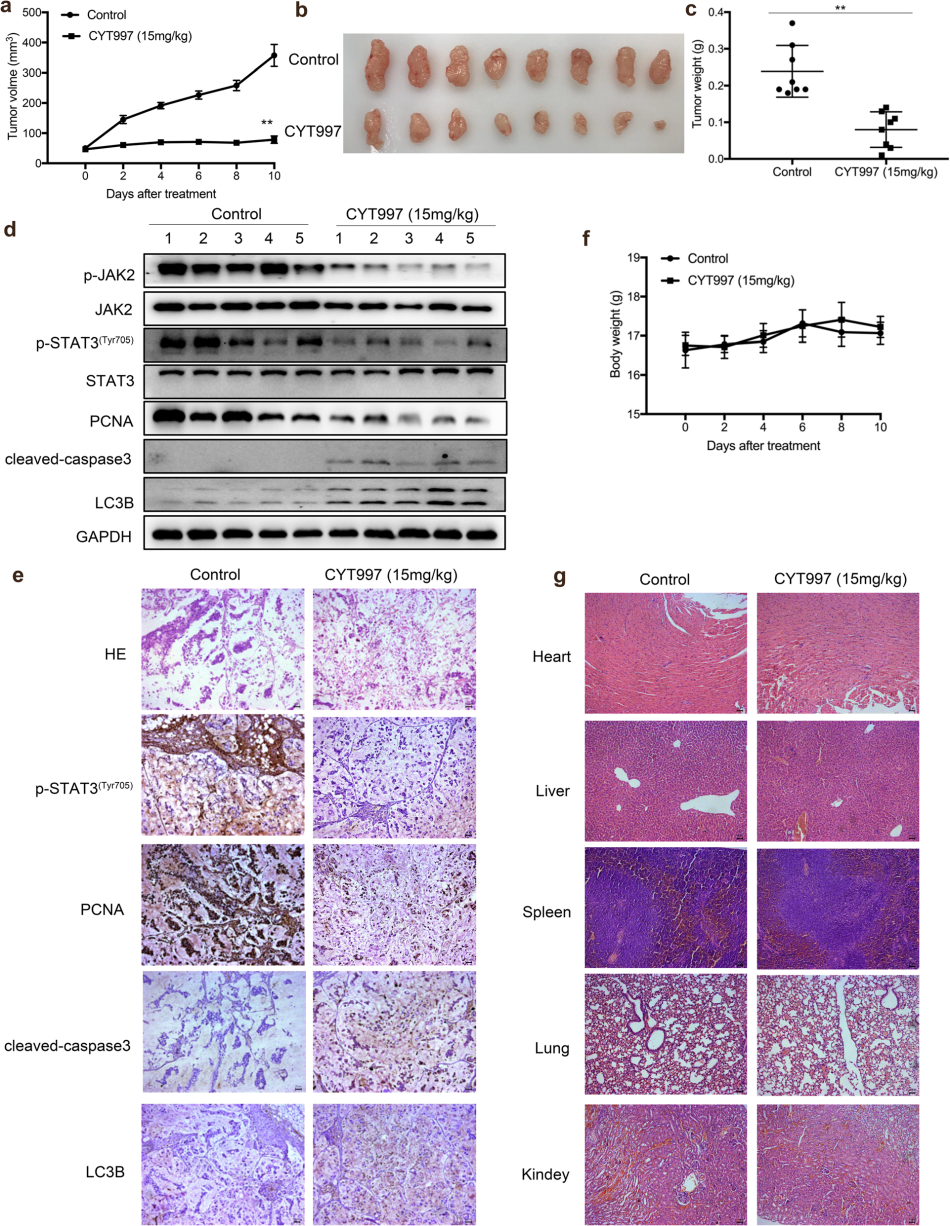
CYT997 suppressed the tumor growth in gastric cancer PDX model. **a** 6 weeks old nude mice with subcutaneous implanted gastric cancer tumor were treated with control or CYT997 for 10 days. Tumor volumes of each group were measured in indicated days of treatment. **p < 0.01. **b** Images of gastric cancer PDX tumor of each group were presented at the same time of study ending. **c** Tumor weights in each group were measured at the end of the treatment and presented. **p < 0.01. **d** The expressions of p-JAK2, JAK2, p-STAT3, STAT3, PCNA and cleaved caspase 3 and LC3B was detected by western blotting in gastric cancer PDX tumor tissues. **E** The expression of p-STAT3, PCNA and cleaved caspase 3 and LC3B was detected by IHC in gastric cancer PDX tumor tissues. Scale bars = 50 μm. **f** Body weights of mice were recorded every other day. **g** HE staining of different tissues. Scale bars = 50 μm

To investigate the effect of CYT997 in tumor growth in vivo, mice bearing gastric cancer PDX xenografts were used. When tumors reached a volume of 50 mm^3^, mice were intraperitoneally injected with normal saline (NS) and CYT997 (15 mg/kg) respectively. Tumor size was measured every other day and tumors were excised after 10 days post injection. We found that CYT997 significantly decreased tumor volume and tumor weight compared with control group (Fig. 7a-c). Furthermore, we also found that the expressions of p-JAK2 and p-STAT3 were decreased after CYT997 treatment. And CYT997 increased the expression of cleaved caspase 3 and LC3B (Fig. 7d-e; Additional file 1: Fig. S9). In addition, we found that there were no obvious changes in body weight and organ-related toxicities were scarce in mice (Fig. 7f and g). Collectively, our results suggest that CYT997 inhibits tumor growth and cell proliferation in vivo.

### CYT997 promotes primary GC cell apoptosis

Primary GC cells were extracted from a GC patient’s tumor tissue and observed by light microscope after CYT997 treatment for 24 h (Additional file 1: Fig. S10a). CYT997 significantly induced cell apoptosis. We found that expression of cleaved caspase 3 and LC3B was upregulated, whereas expression of p-JAK2 and p-STAT3 was downregulated in CYT997-treated primary GC cells (Additional file 1: Fig. S10b-10c). Furthermore, ROS scavenger NAC also distinctly weakened CYT997-induced cell apoptosis (Additional file 1: Fig. S10d). CYT997-induced inhibition of p-JAK2, p-STAT3 was reversed by NAC in primary GC cells (Additional file 1: Fig. S10e). Therefore, these data suggested that CYT997 promoted GC cell apoptosis by inhibiting JAK2/STAT3 pathway.

## Discussion

Microtubule targeting agents disrupting the normal function of the mitotic spindle have been proven to be one of the main chemotherapy treatments in GC [9]. Microtubules exert a critical role in cellular functions, such as chromosome segregation during cell division, intracellular transport, cell motility, and the maintenance of cell shape [31]. Microtubule targeting agents disrupt the tubulin dynamics by binding to the distinct sites on protein tubulin and block their polymerization dynamics, causing mitotic spindle disorder, resulting in prolonged mitotic arrest, subsequent growth arrest and cell death [8, 32]. Therefore, finding new microtubule-targeting drugs is important in GC therapy. CYT997, as a novel synthetic microtubule-disrupting agent, its function in GC treatment hasn’t been deeply explored yet. In our study, we explored CYT997 efficacy in treating GC and found CYT997 inhibited cell proliferation, induced G2/M phase arrest, triggered apoptosis and autophagy in GC cells. More importantly, we further elucidated the underlying antitumor mechanisms of CYT997 in GC and proved that CYT997 could suppress GC cell growth and induce apoptosis through upregulating ROS product to inhibit activation of STAT3 signaling pathway. Meanwhile, CYT997 could trigger autophagy to prevent GC cells apoptosis, suggesting that the combination therapy of CYT997 and autophagy inhibitor in GC could achieve a better antitumor efficacy than separate application.

Normally, ROS are formed in oxygen metabolism and mainly produced by mitochondrial redox chain [33, 34]. During normal physiological metabolism of tumors, elevated ROS can be regulated to keep a balance by antioxidant systems to maintain tumor cell survival. ROS are critical signaling molecules, exerting important function in promoting cell autophagy and apoptosis [25, 35, 36]. Antitumor drugs such as docetaxel, cisplatin often break the balance of oxidant and antioxidant systems, damage mitochondrial membrane potential, increase ROS production and silence some signaling pathways such as Akt/mTOR signaling pathway to lead to tumor cell apoptosis [37–39]. In this study, we found that CYT997 induced GC cell apoptosis through increasing mitochondrial ROS production, suggesting that CYT997 may be a promising antitumor drug. The ROS scavenger, NAC and MitoQ, distinctly weakened the effects of CYT997 on GC cells. Therefore, mitochondrial ROS exert a critical role in CYT997-induced GC cells apoptosis.

STAT3 (Signal transducer and activator of transcription 3), a critical transcriptional factor of tumorigenesis and a point of convergence of most activated oncogenic pathways, plays a pivotal role in tumor initiation and development [40, 41]. Activated STAT3 was found in diverse cancers, such as GC, promoting tumor cell growth, proliferation, anti-apoptosis, cancer angiogenesis and metastasis [30]. Some antitumor drugs, such as targeting microtubule paclitaxel, have been proved to inhibit activation of STAT3 to induce tumor cells apoptosis [42]. Given its fundamental role in tumor proliferation and progression, STAT3 has emerged as a promising target for cancer treatment, especially in GC therapy [43]. Furthermore, related researches have proved that upregulated ROS could abrogate JAK2/STAT3 signaling to suppress tumor growth in cancers such as hepatocellular carcinoma [44], multiple myeloma [45], and colorectal cancer [46, 47]. Therefore, activated ROS production played a key role in suppression of JAK2/STAT3 signaling. In our study, we found that CYT997 remarkably suppressed expression of p-JAK2 and p-STAT3 in GC cells. Furthermore, CYT997 suppressed IL-6-induced STAT3 activation. In addition, overexpression of STAT3 significantly attenuated the effect of CYT997 on GC cells. We found that ROS triggered by CYT997 inhibited JAK2/STAT3 pathway in GC cells. Therefore, all results indicated that CYT997 inhibited cell proliferation and induced apoptosis by regulating of JAK2/STAT3 pathway in GC cells.

## Conclusion

**Fig. 8:**
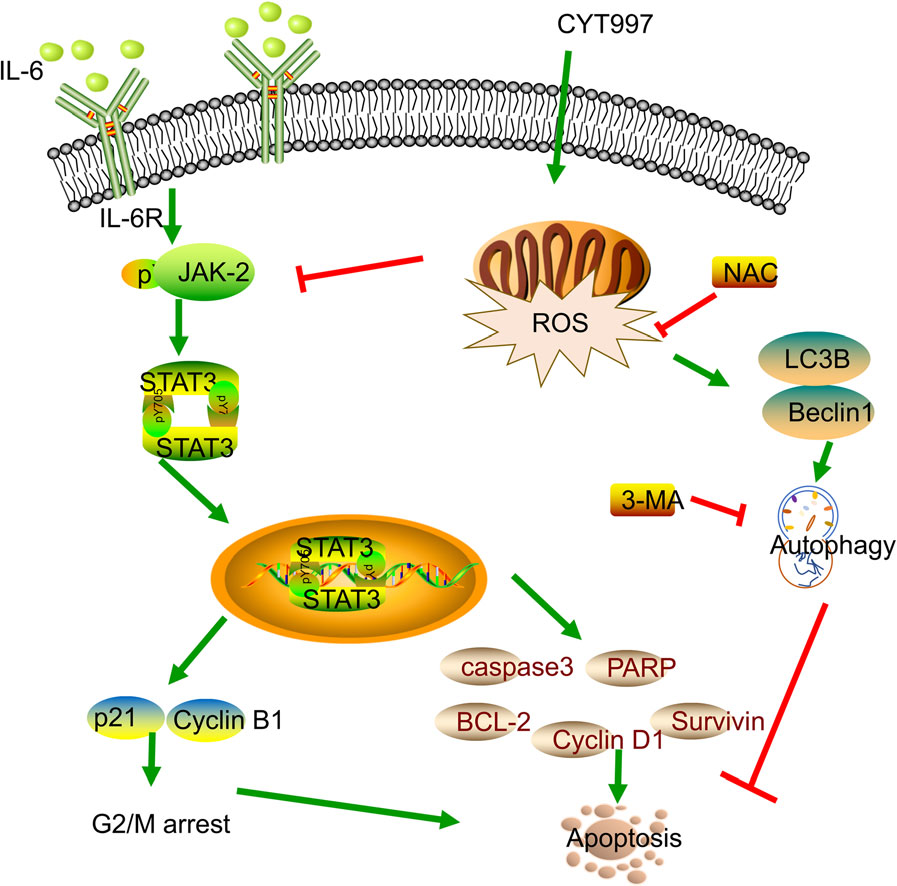
Mechanism of CYT997-induced apoptosis in GC cells. CYT997 activated mitochondrial ROS to trigger protective autophagy and inhibited JAK2/STAT3 pathway to induce G2/M arrest and apoptosis in GC

Our research for the first time showed that CYT997 suppressed GC proliferation and promoted apoptosis by the regulation of ROS/JAK2/STAT3 signaling pathways (Fig.8). Our study indicates that CYT997 might be a potential antitumor drug candidate to treat gastric cancer.

## Supplementary information

**Additional file 1: Fig. S1.** CYT997 inhibited human osteosarcoma 143B cells proliferation. *p < 0.05; **p < 0.01. **Fig. S2.** Quantification analysis of western blot images. a and b corresponded to Fig. 2b and Fig. 2d respectively. **p < 0.01. **Fig. S3.** Quantification analysis of fluorescence intensity and western blot images. **a** Lysotracker in Fig. 3a was quantified. Fold of lysotracker intensity in GC cells treated with CYT997 was significantly high. **b** LC3-GFP in Fig. 3a was quantified. Fold of LC3-GFP intensity in GC cells treated with CYT997 was significantly high. **c** Western blot images in in Fig. 3b was quantified. **d** Western blot images in Fig. 3d was quantified. The results shown here are representative of three independent experiments. **p < 0.01. **Fig. S4.** Protein expression level of Fig. 4i. **p < 0.01. **Fig. S5.** Quantification analysis of western blot images and the effect of MitoQ. **a** Protein expression level of Fig. 5d. **b **SGC-7901 cells were treated with CYT997 or in combination with MitoQ (500 nM). The expression of p-JAK2, JAK2, p-STAT3, STAT3 was detected by western blotting in SGC-7901 cells. **p < 0.01. **Fig. S6.** Quantification analysis of western blot images. **a**, **b** and **c** corresponded to Fig. 5a, Fig. 5b and Fig. 5c respectively. **p < 0.01. **Fig. S7.** Quantification analysis of western blot images. **a**, **b** and **c** corresponded to Fig. 5e, Fig. 5i and Fig. 5j respectively. **p < 0.01. **Fig. S8.** Overexpression JAK2 in GC cells could reversed inhibition of CYT997.**a-d** SGC-7901 cells were transfected with JAK2 vector, and then treated with CYT997. Cell viability was detected by a CCK8 assay (**a**). The cell cycle distribution was analyzed by flow cytometry (**b**). Apoptosis was detected by flow cytometry (**c**). The expression of JAK2, p-JAK2, Cyclin B1, p21, cleaved PARP and cleaved caspase 3 was detected by western blotting. **p < 0.01. **Fig. S9.** Protein expression level of Fig. 6d. **p < 0.01. **Fig. S10.** CYT997 promoted primary cells apoptosis. **a-e** Primary cells were extracted from a GC patient’s tumor tissue, and then treated with CYT997. Cells morphology was changed after CYT997 treatment for 24 h. Scale bars =100 μm (**a**). Apoptosis was detected by flow cytometry (**b**). The expression of p-JAK2, JAK2, p-STAT3, STAT3, cleaved caspase 3 and LC3B was detected by western blotting (**c**). Apoptosis was detected by flow cytometry after CYT997 treatment alone or in combination with NAC (5 mM) (**d**). The expression of p-JAK2, JAK2, p-STAT3, STAT3, cleaved caspase 3 and LC3B was detected by western blotting after CYT997 treatment alone or in combination with NAC (5 mM) (**e**). **p < 0.01.

## Data Availability

All data generated or analyzed during this study are available from the corresponding author upon reasonable request.

## Abbreviations

GC: Gastric cancer
ROS: Reactive oxygen species
STAT3: Signal transducer and activator of transcription 3
p-STAT3: Phosphorylated STAT3
p-JAK2: Phosphorylated JAK2
NAC: N-acetyl-L-cysteine
MitoQ: Mitoquinone
3-MA: 3-methyladenine
FBS: Fetal bovine serum
PBS: Phosphate-buffered saline
