# Supplementary material for: Mitochondrial ROS accumulation inhibiting JAK2/STAT3 pathway is a critical modulator of CYT997-induced autophagy and apoptosis in gastric cancer
Source: J Exp Clin Cancer Res. 2020 Jun 23;39:119. doi: 10.1186/s13046-020-01621-y (PMC7310559; doi:10.1186/s13046-020-01621-y)
Supplement: Supplementary file 1 — Additional file 1: Fig. S1. CYT997 inhibited human osteosarcoma 143B cells proliferation. *p < 0.05; **p < 0.01. Fig. S2. Quantification analysis of western blot images. a and b corresponded to Fig. 2b and Fig. 2d respectively. **p < 0.01. Fig. S3. Quantification analysis of fluorescence intensity and western blot images. a Lysotracker in Fig. 3a was quantified. Fold of lysotracker intensity in GC cells treated with CYT997 was significantly high. b LC3-GFP in Fig. 3a was quantified. Fold of LC3-GFP intensity in GC cells treated with CYT997 was significantly high. c Western blot images in in Fig. 3b was quantified. d Western blot images in Fig. 3d was quantified. The results shown here are representative of three independent experiments. **p < 0.01. Fig. S4. Protein expression level of Fig. 4i. **p < 0.01. Fig. S5. Quantification analysis of western blot images and the effect of MitoQ. a Protein expression level of Fig. 5d. b SGC-7901 cells were treated with CYT997 or in combination with MitoQ (500 nM). The expression of p-JAK2, JAK2, p-STAT3, STAT3 was detected by western blotting in SGC-7901 cells. **p < 0.01. Fig. S6. Quantification analysis of western blot images. a, b and c corresponded to Fig. 5a, Fig. 5b and Fig. 5c respectively. **p < 0.01. Fig. S7. Quantification analysis of western blot images. a, b and c corresponded to Fig. 5e, Fig. 5i and Fig. 5j respectively. **p < 0.01. Fig. S8. Overexpression JAK2 in GC cells could reversed inhibition of CYT997.a-d SGC-7901 cells were transfected with JAK2 vector, and then treated with CYT997. Cell viability was detected by a CCK8 assay (a). The cell cycle distribution was analyzed by flow cytometry (b). Apoptosis was detected by flow cytometry (c). The expression of JAK2, p-JAK2, Cyclin B1, p21, cleaved PARP and cleaved caspase 3 was detected by western blotting. **p < 0.01. Fig. S9. Protein expression level of Fig. 6d. **p < 0.01. Fig. S10. CYT997 promoted primary cells apoptosis. a-e Primary cells were extracted fr [file 13046_2020_1621_MOESM1_ESM.docx]

**Mitochondrial ROS accumulation inhibiting JAK2/STAT3 pathway is a critical modulator of CYT997-induced autophagy and apoptosis in gastric cancer**

Ya Cao^1, 2^, Jinglong Wang^1^, Hua Tian^2*^,Guo-Hui Fu^1*^

^1^Pathology Center, Shanghai General Hospital/Faculty of Basic Medicine, Key Laboratory of Cell Differentiation and Apoptosis of Chinese Ministry of Education, Institutes of Medical Sciences, Shanghai Key Laboratory of Gastric Neoplasms, Shanghai Institute of Digestive Surgery, Ruijin Hospital, Shanghai Jiao Tong University School of Medicine, Shanghai, China
^2^ State Key Laboratory of Oncogenes and Related Genes, Shanghai Cancer Institute, Renji Hospital, Shanghai Jiaotong University School of Medicine, Shanghai, China

*Correspondence: *Guo-Hui Fu, Ph.D., Pathology Center, Shanghai General Hospital/Faculty of Basic Medicine, Key Laboratory of Cell Differentiation and Apoptosis of Chinese Ministry of Education, Institutes of Medical Sciences, Shanghai Key Laboratory of Gastric Neoplasms, Shanghai Institute of Digestive Surgery, Ruijin Hospital, Shanghai Jiao Tong University School of Medicine No. 280, South Chong-Qing Road, Shanghai, 200025, China. E-mail: fuguhu@263.net.*

*Hua Tian, Ph.D., State Key Laboratory of Oncogenes and Related Genes, Shanghai Cancer Institute, Renji Hospital, Shanghai Jiaotong University School of Medicine, 25/Ln 2200, Xietu Road, Shanghai 200032, China. Tel/Fax: +86-21-64436627，E-mail:**[htian@shsci.org](mailto:htian@shsci.org)*


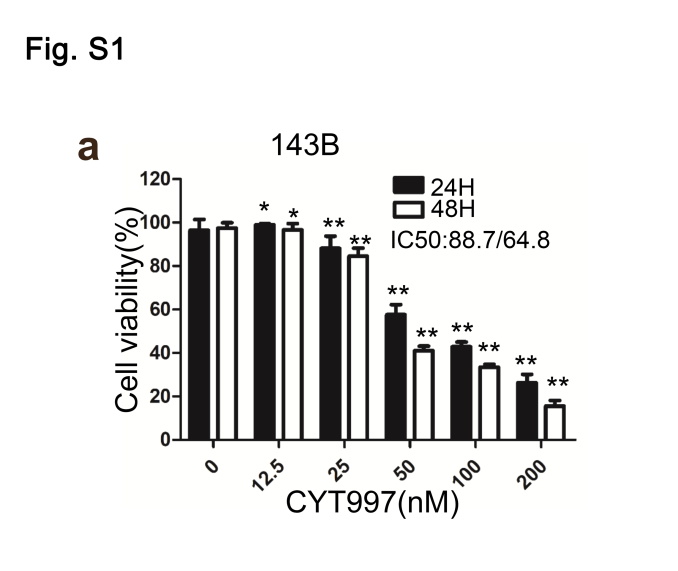


**Fig. S1** CYT997 inhibited human osteosarcoma 143B cells proliferation.*p< 0.05; **p< 0.01.


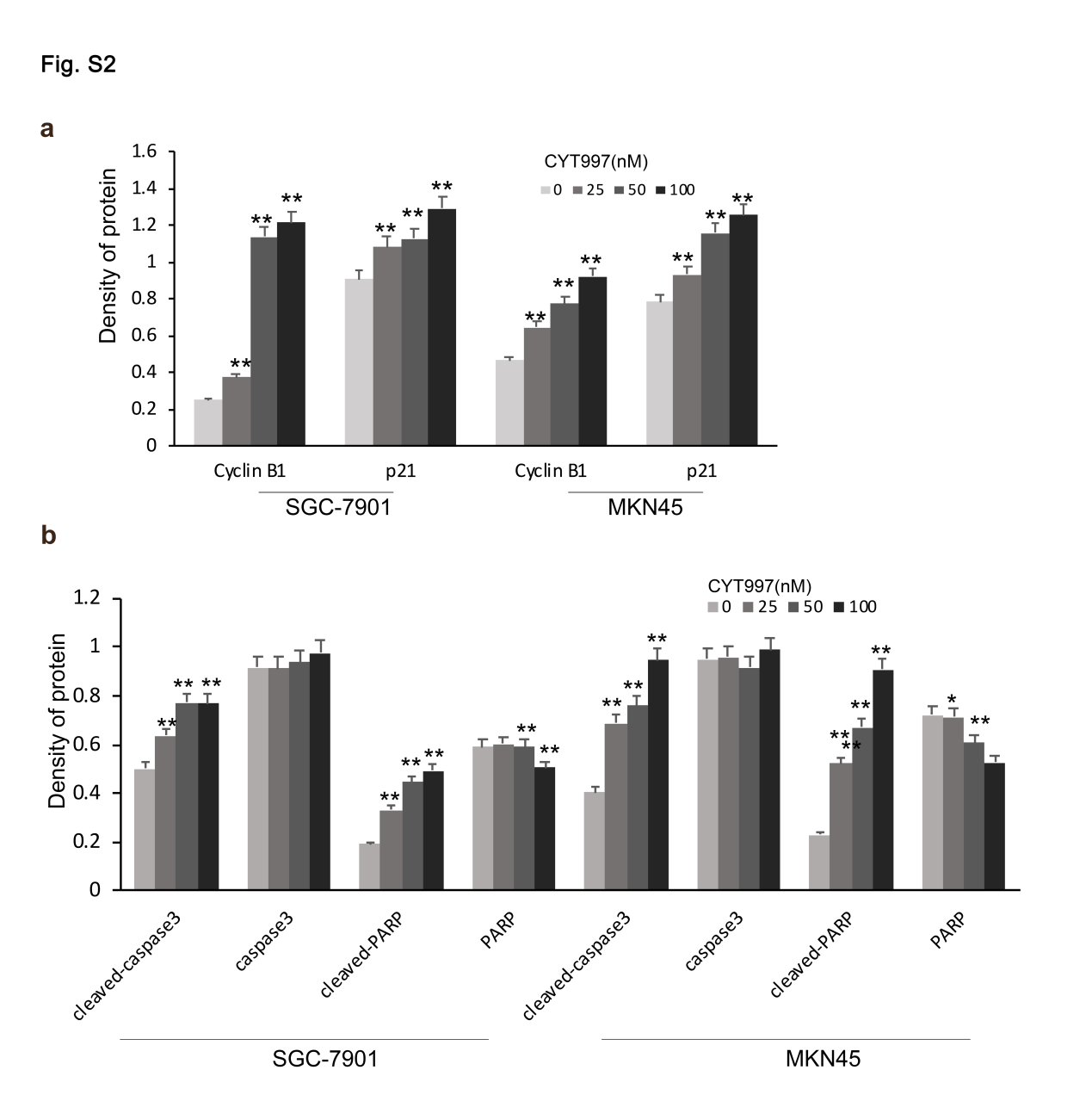


**Fig. S2** Quantification analysis of western blot images. a and b corresponded to Fig 2b and Fig 2d respectively. **p< 0.01.

**
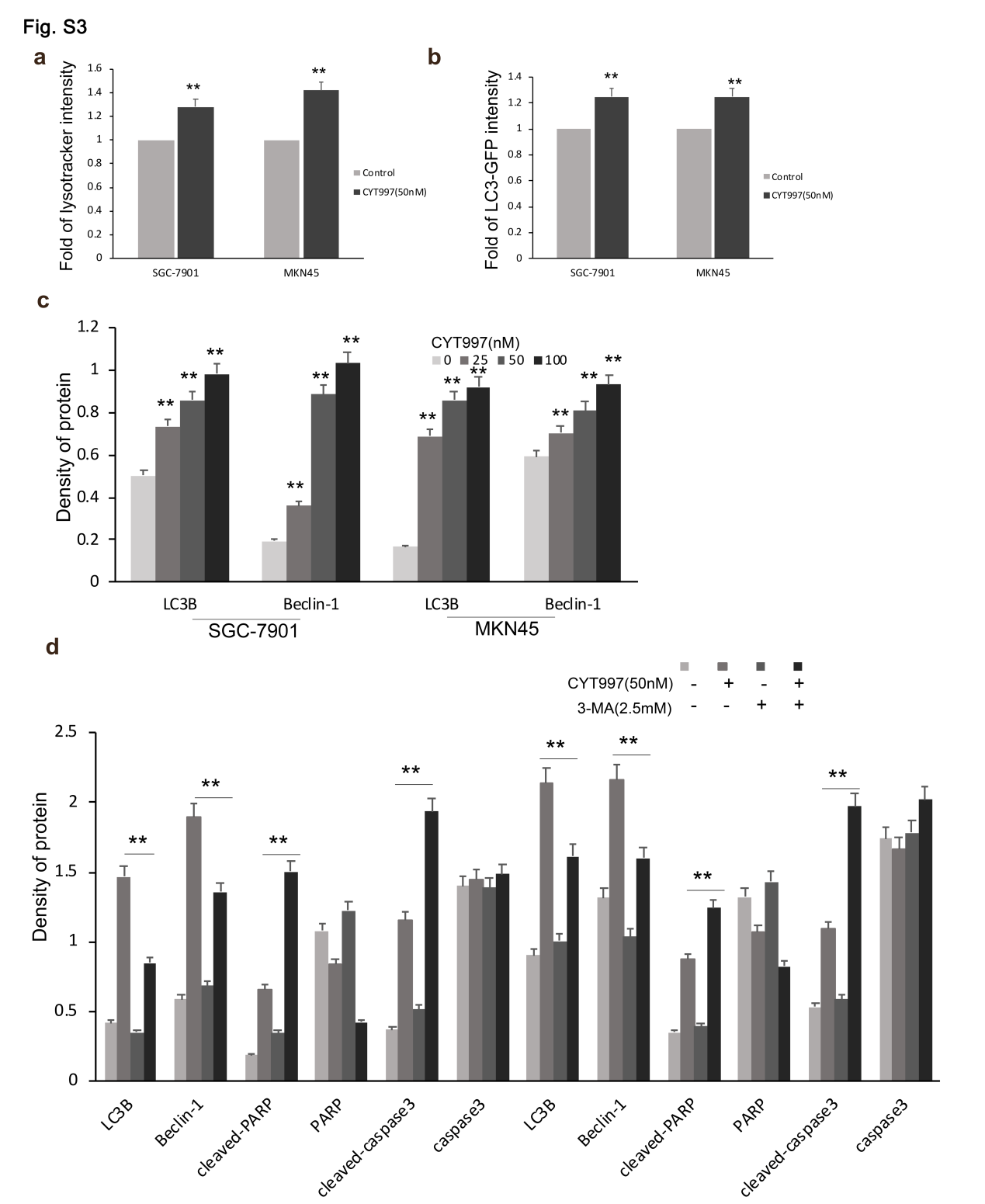
**

**Fig. S3** Quantification analysis of fluorescence intensity andwestern blot images. **a** Lysotracker in Fig 3a was quantified. Fold of lysotracker intensity in GC cells treated with CYT997 was significantly high.**B**LC3-GFP in Fig 3a was quantified. Fold of LC3-GFP intensity in GC cells treated with CYT997 was significantly high.**c** Western blot images in in Fig 3b was quantified.**d** Western blot images in Fig 3d was quantified. The results shown here are representative of three independent experiments. **p< 0.01.


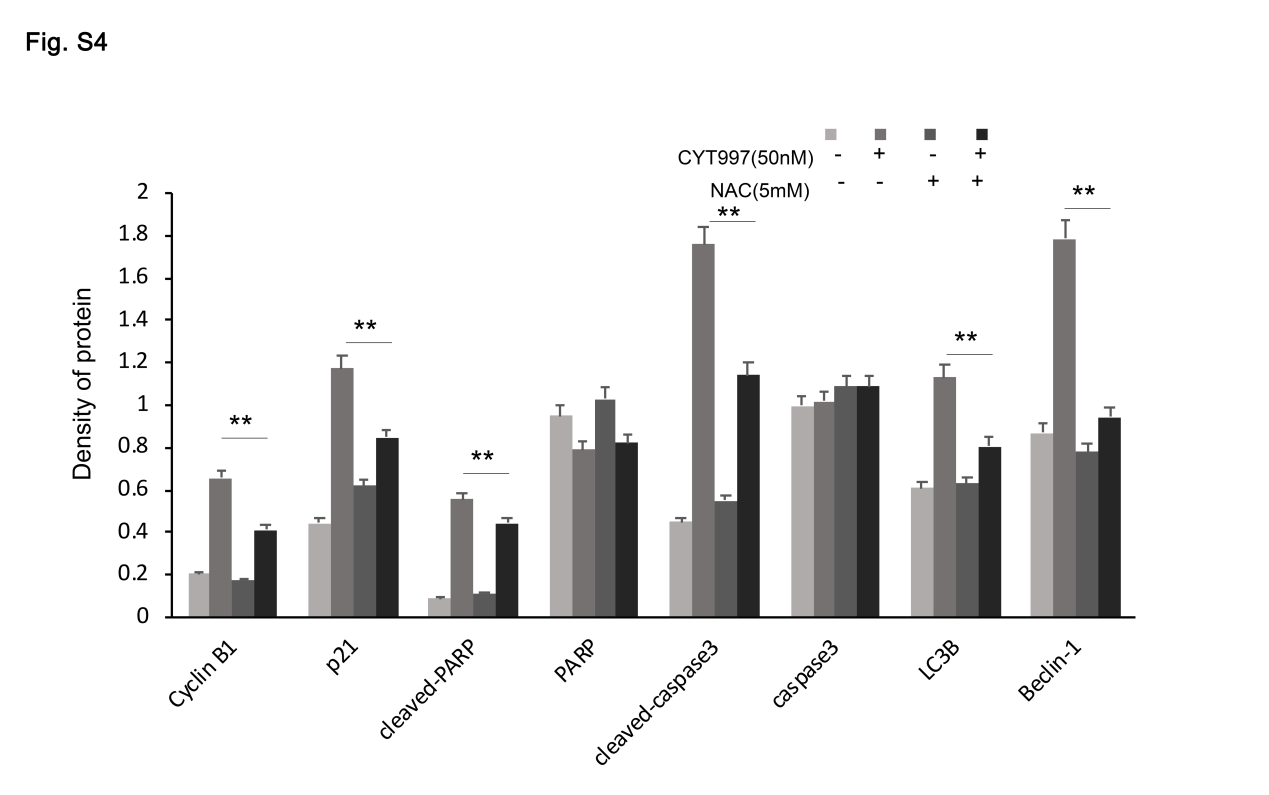


**Fig. S4** Protein expression level of Fig 4i. **p< 0.01.


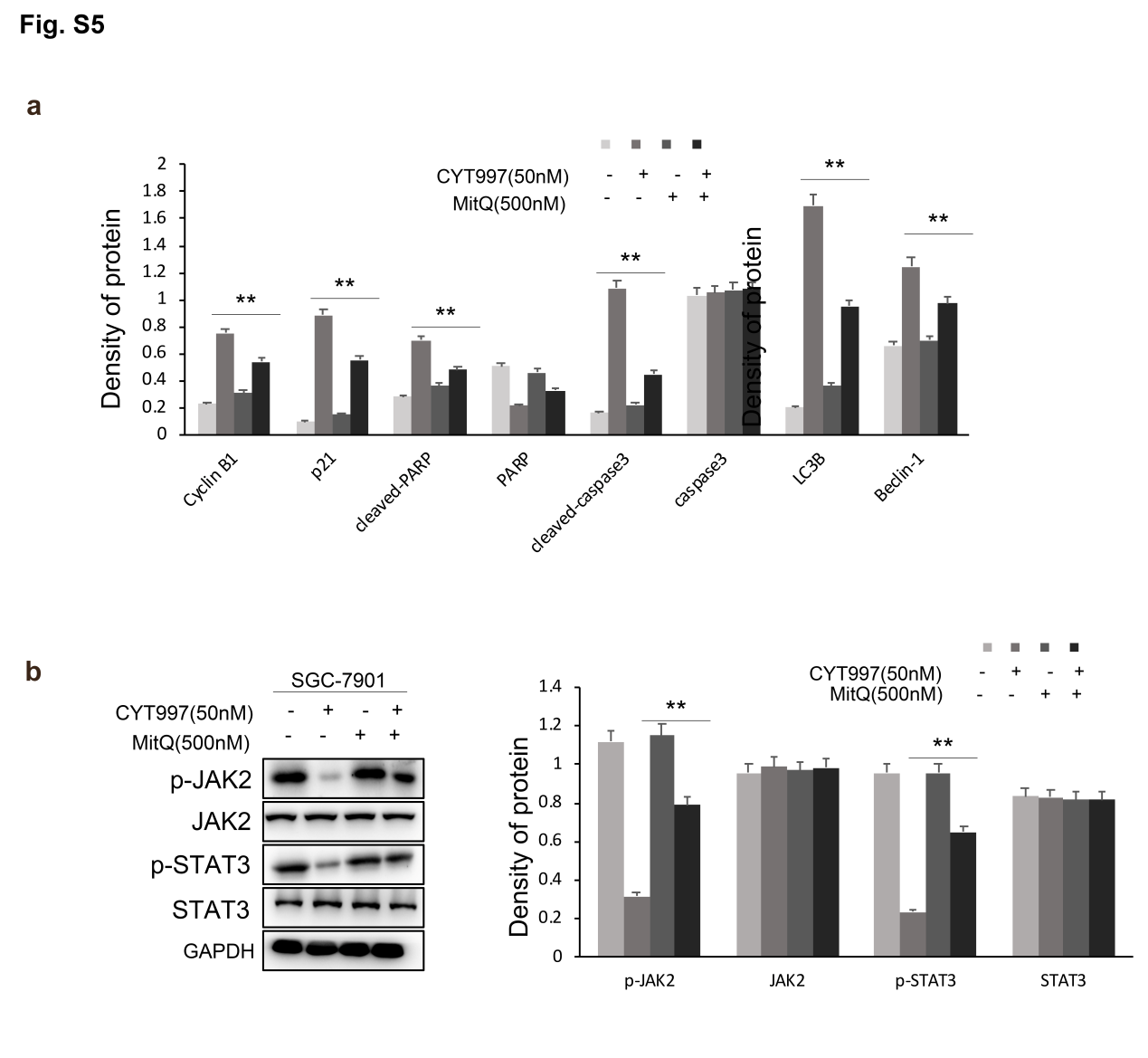


**Fig.S5** Quantification analysis of western blot imagesand the effect of MitoQ. **a** Protein expression level of Fig 5d.**b** SGC-7901 cells were treated with CYT997 or in combination with MitoQ (500nM). The expression of p-JAK2, JAK2, p-STAT3, STAT3 was detected by western blotting in SGC-7901 cells. **p< 0.01.


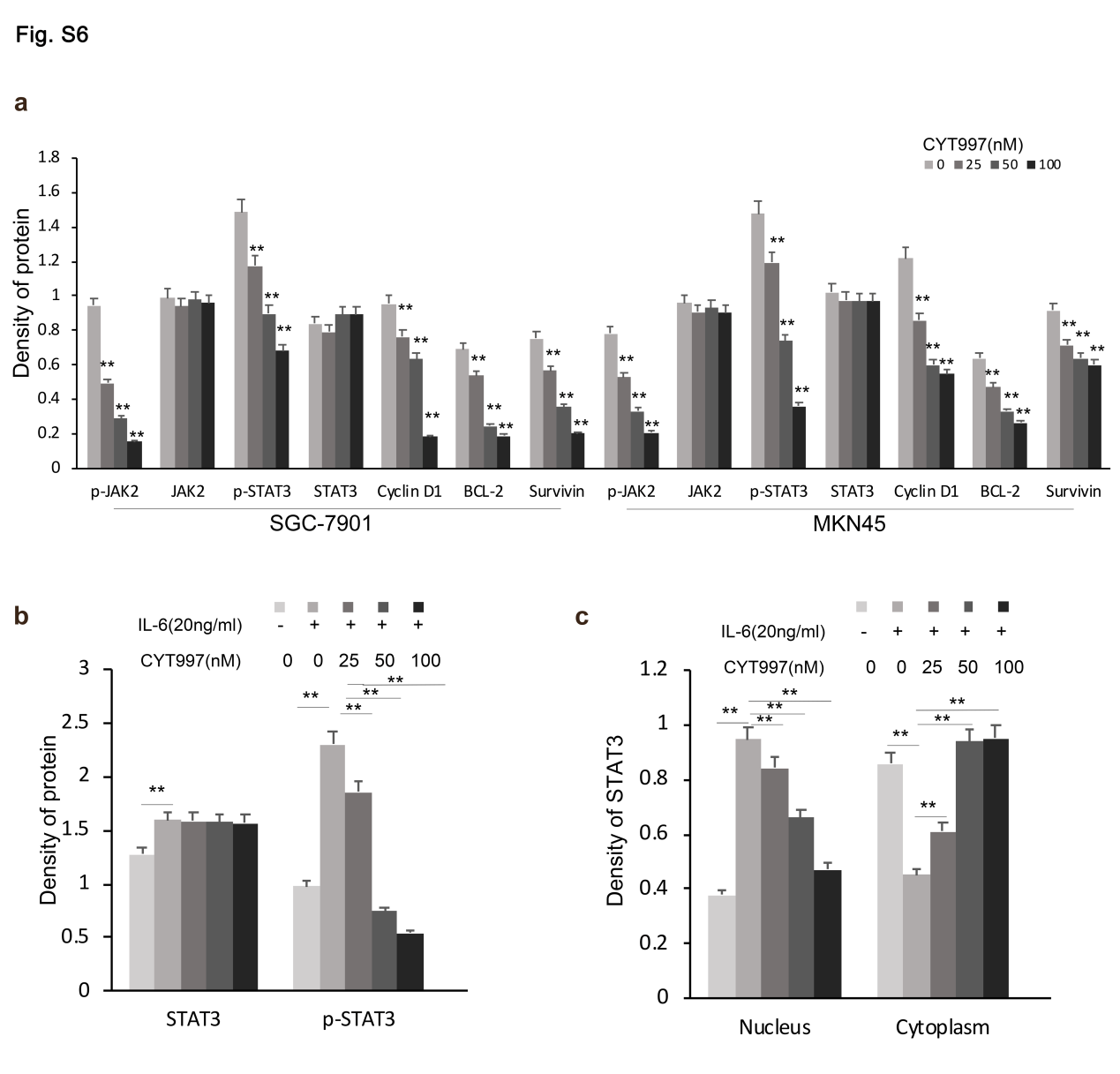


**Fig. S6** Quantification analysis of western blot images. **a**, **b** and **c** corresponded to Fig 5a, Fig 5b and Fig 5c respectively. **p< 0.01.


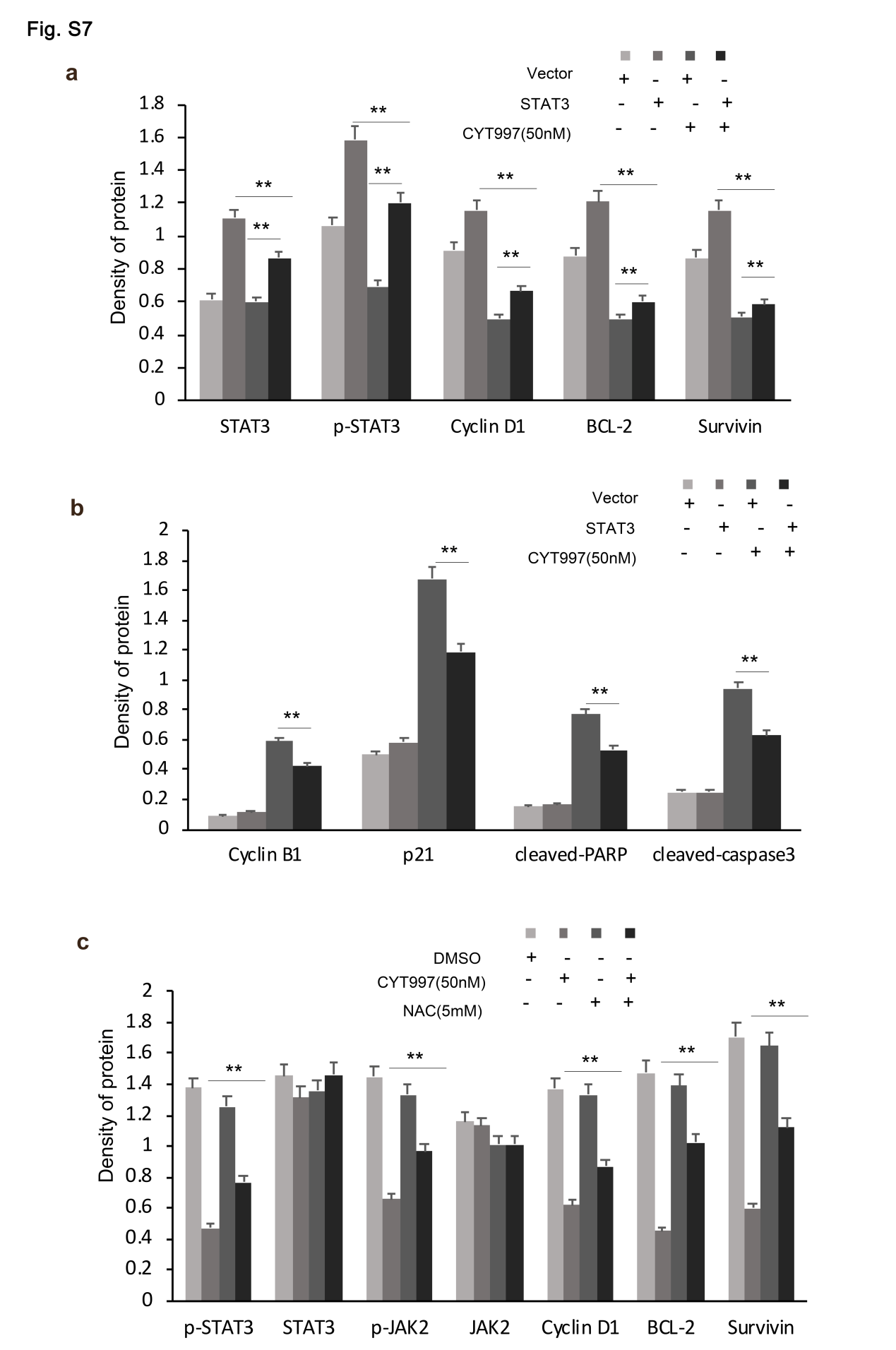


**Fig. S7** Quantification analysis of western blot images. **a**, **b** and **c** corresponded to Fig 5e, Fig 5i and Fig 5j respectively. **p< 0.01.


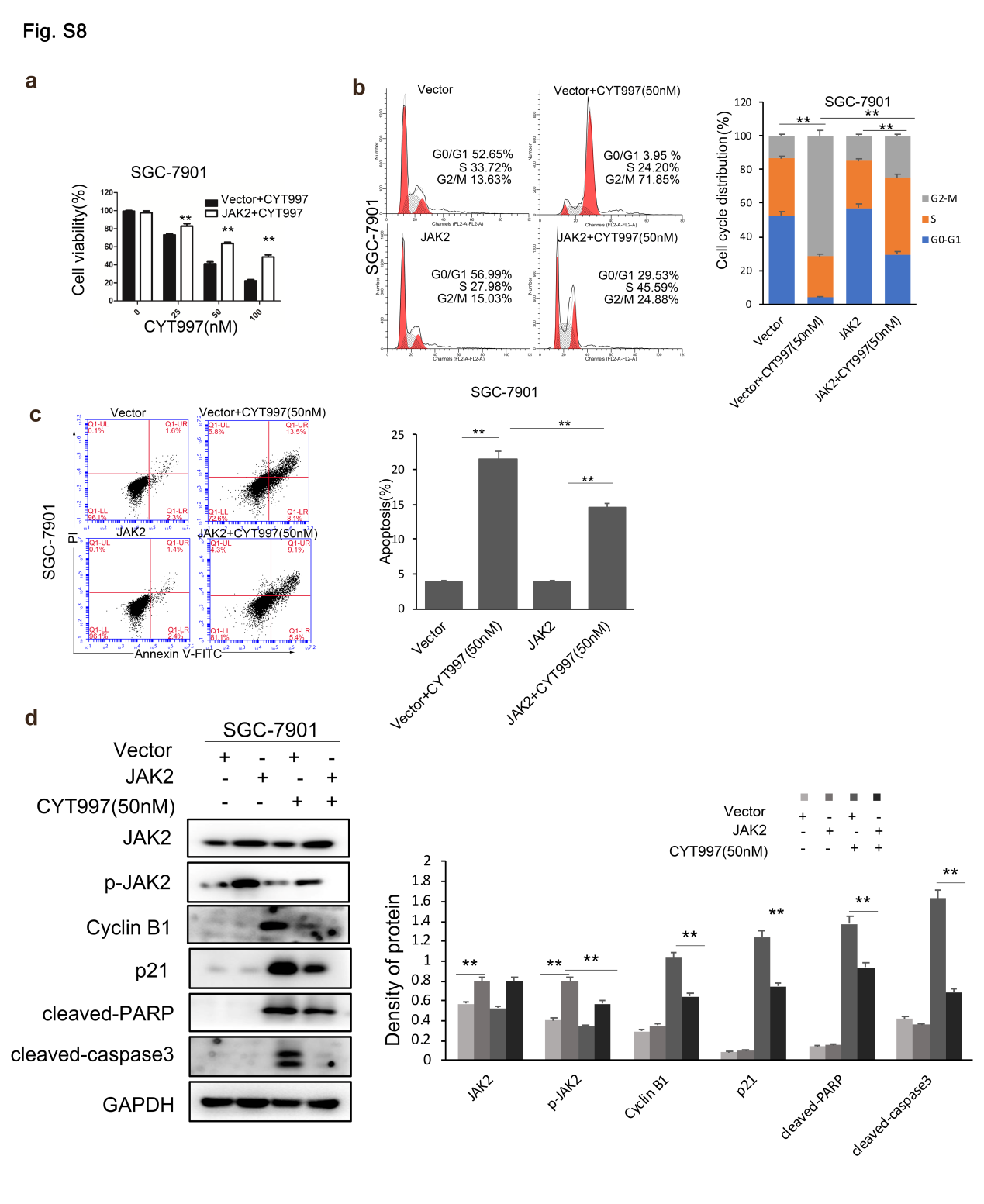


**Fig. S8** Overexpression JAK2 in GC cells could reversed inhibition of CYT997.**a-d** SGC-7901 cells were transfected with JAK2 vector, and then treated with CYT997. Cell viability was detected by a CCK8 assay (**a**). The cell cycle distribution was analyzed byflow cytometry (**b**).Apoptosis was detected by flow cytometry(**c**). The expression of JAK2, p-JAK2, Cyclin B1, p21, cleaved PARP and cleaved caspase 3 was detected by western blotting. **p< 0.01.


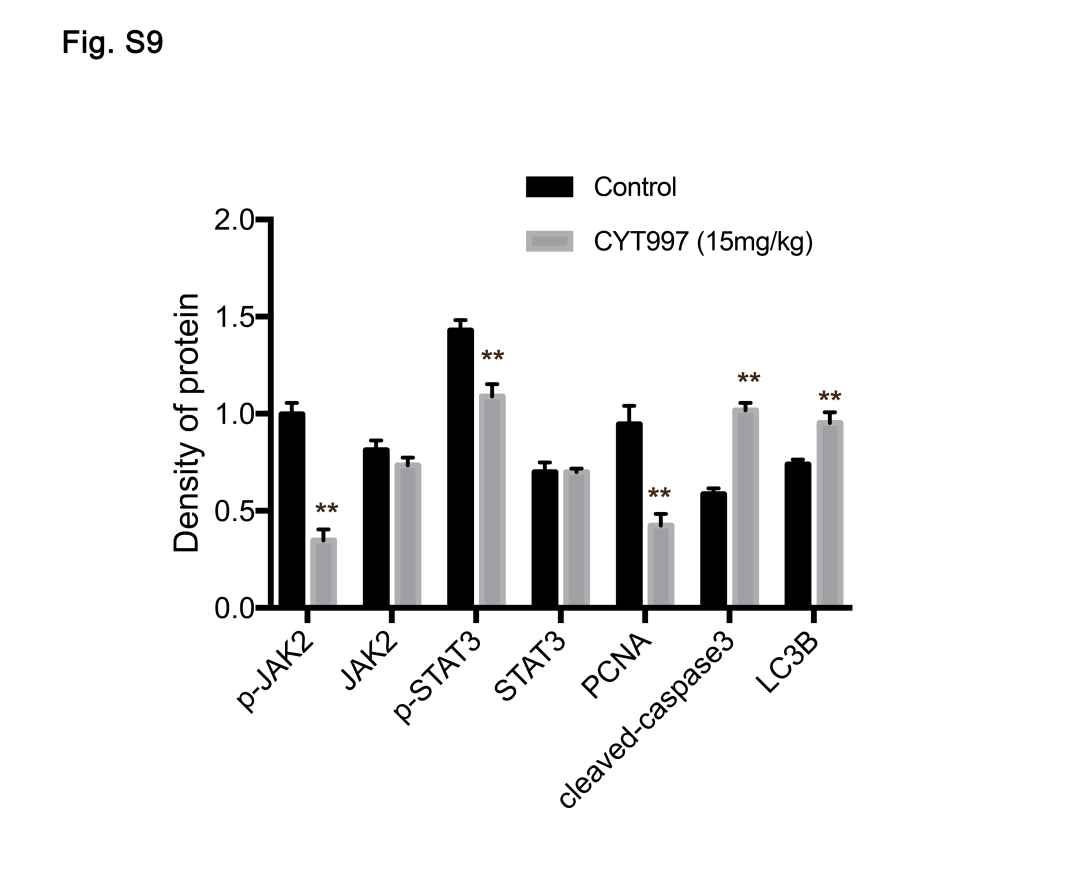


**Fig. S9** Protein expression level of Fig 6d. **p< 0.01.


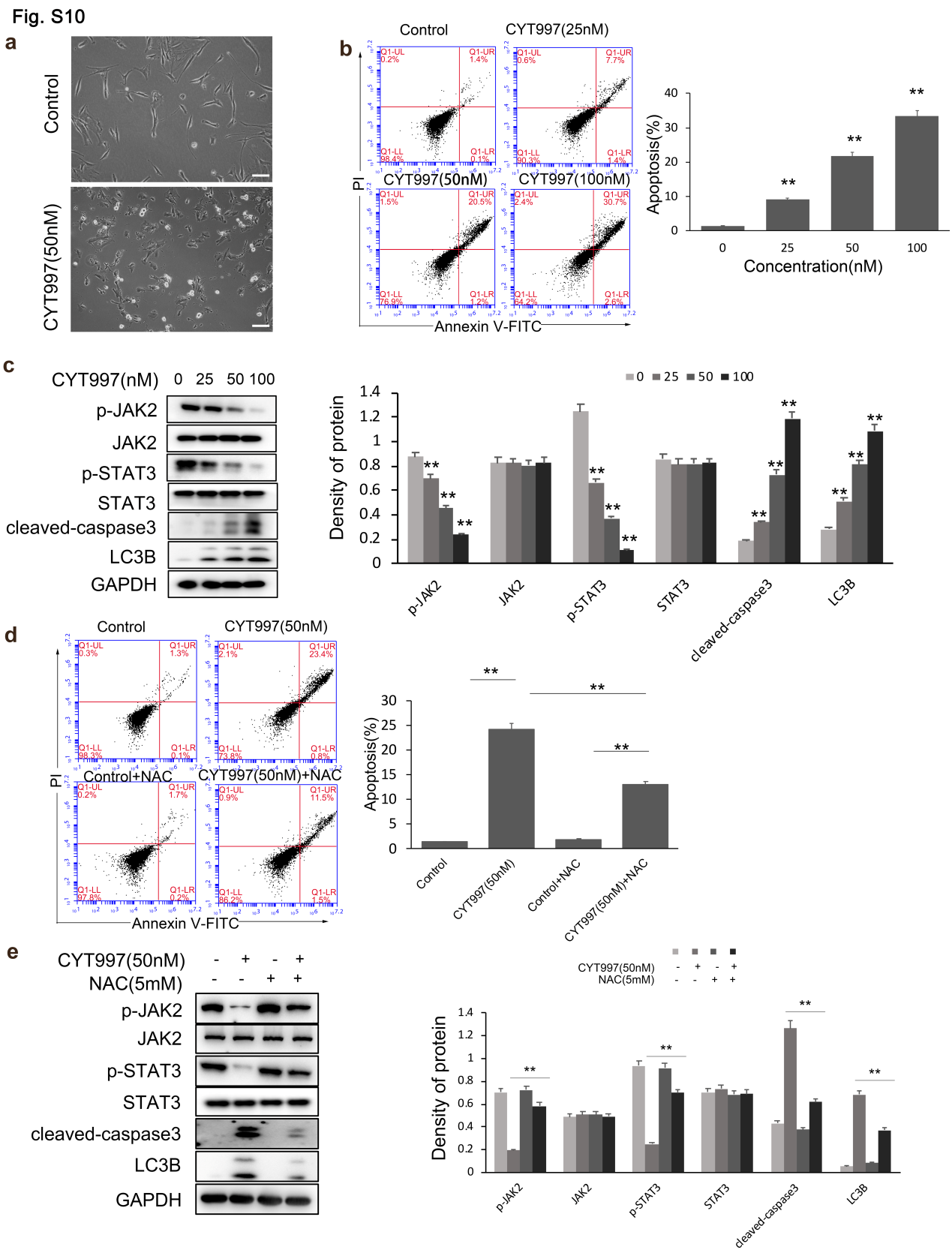


**Fig. S10** CYT997 promoted primary cells apoptosis.**a-e** Primary cells were extracted from a GC patient’s tumor tissue, and then treated with CYT997. cells morphology was changed after CYT997 treatment for 24 h. Scale bars =100µm(**a**). Apoptosis was detected by flow cytometry (**b**).The expression of p-JAK2, JAK2, p-STAT3, STAT3, cleaved caspase 3 and LC3B was detected by western blotting (**c**). Apoptosis was detected by flow cytometry after CYT997 treatment alone or in combination with NAC (5mM)(**d**). The expression of p-JAK2, JAK2, p-STAT3, STAT3, cleaved caspase 3 and LC3B was detected by western blotting after CYT997 treatment alone or in combination with NAC (5mM) (**e**). **p< 0.01.
